# Naïve CD4^+^ cell counts significantly decay and high HIV RNA levels contribute to immunological progression in long-term non-progressors infected with HIV by blood products: a cohort study

**DOI:** 10.1186/s12865-021-00426-8

**Published:** 2021-06-03

**Authors:** Ling Xu, Yubin Liu, Xiaojing Song, Yanling Li, Yang Han, Ting Zhu, Wei Cao, Taisheng Li

**Affiliations:** 1 Department of Infectious Diseases, Peking Union Medical College Hospital, Peking Union Medical College and Chinese Academy of Medical Sciences, Beijing, China; 2 Clinical Immunology Center, Chinese Academy of Medical Sciences, Beijing, China; 3 Tsinghua University Medical College, No. 1 Shuaifuyuan, Wangfujing Street, Beijing, 100730 China

**Keywords:** LTNPs, Blood products, naïve CD4^+^ T cell count diminishing, High HIV RNA levels, Immune activation

## Abstract

**Background:**

Some long-term non-progressors (LTNPs) have decreasing CD4^+^ T cell counts and progress to AIDS. Exploring which subsets of CD4^+^ T cell decreasing and the determinants associated with the decay in these patients will improve disease progression surveillance and provide further understanding of HIV pathogenesis.

**Methods:**

Twenty-five LTNPs infected with HIV by blood products were classified as decreased (DG) if their CD4^+^ cell count dropped to < 400 cells/μL during follow-up or as non-decreased (non-DG) if their CD4^+^ cell count was ≥400 cells/μL. Laboratory and clinical assessments were conducted at 6 consecutive visits to identify DG characteristics.

**Results:**

The LTNPs were infected with HIV for 12 (IQR: 11.5–14) years, and 23 were classified as the B′ subtype. Six individuals lost LTNP status 14.5 (IQR: 12.5–17.5) years after infection (DG), and the CD4^+^ T cell count decreased to 237 (IQR: 213–320) cells/μL at the latest visit. The naïve CD4^+^ T cell count decrease was greater than that of memory CD4^+^ T cells [− 128 (IQR: − 196, − 107) vs − 64 (IQR: − 182, − 25) cells/μL)]. Nineteen individuals retained LTNP status (non-DG). At enrolment, the viral load (VL) level (*p* = 0.03) and CD8^+^CD38^+^ percentage (*p* = 0.03) were higher in DG than non-DG individuals. During follow-up, viral load and CD8^+^CD38^+^ percentage were significantly increased and negatively associated with CD4^+^ cell count [(r = − 0.529, *p* = 0.008), (r = − 0.476, *p* = 0.019), respectively]. However, the CD8^+^CD28^+^ percentage and B cell count dropped in DG and were positively correlated with CD4^+^ T cell count [(r = 0.448, *p* = 0.028), (r = 0.785, *p* < 0.001)].

**Conclusion:**

Immunological progression was mainly characterized by the decrease of naïve CD4^+^ T cell in LTNPs infected with HIV by blood products and it may be associated with high HIV RNA levels.

**Supplementary Information:**

The online version contains supplementary material available at 10.1186/s12865-021-00426-8.

## Introduction

There were 38 million people living with HIV-1 worldwide in 2019 (https://www.unaids.org). HIV-infected individuals are characterized by continuous HIV RNA replication and CD4^+^ T cell count decline, and most of them progress to AIDS in an average of 10 years [1]. However, approximately 5–15% of HIV-infected patients can naturally maintain high CD4^+^ T cell counts and remain asymptomatic for several years without cART (combined antiretroviral therapy). These individuals are considered as long-term non-progressors (LTNPs), including elite controllers (ECs), who only account for 1% of the HIV-infected patients [2, 3].

Unfortunately, some LTNPs eventually exhibit immunologic progression with CD4^+^ T cell count decay [4]. After 12.49 years (95% CI: 12.05–12.92) of HIV seroconversion, approximately 53.0% LTNPs experienced a decrease in CD4^+^ T cell count to below 500 cells/μL in a European study [5]. CD4^+^ T cell count is an important indicator of immune function in HIV-infected individuals. Patients with decreasing CD4^+^ T cell counts have been associated with increased risk of various opportunistic infections due to immunosuppression [6].

Previous studies showed that various factors were associated with CD4^+^ T cell count decay in HIV-infected patients. For example, the protective HLA class I allele HLA-B*57 was more frequent among LTNP-controllers, defined by maintaining CD4^+^ T cell counts > 500 cells/mm^3^ for more than 7 years after HIV diagnosis, than among non-LTNP-controllers, defined by CD4^+^ T cell counts dropping to less than 500 cells/mm^3^ [7]. Additionally, elevated anti-CMV titers were associated with disease progression in HIV ECs [8], and IL-32 was considered a predictive biomarker for disease progression in slow progressors [9]. However, these studies were often limited due to a cross-sectional design.

More importantly, it was well known that naïve CD4^+^ T cell counts seriously decreased in chronic HIV-infected individuals [10, 11], data on LTNPs was limited owing to the limitation of the analyses to total CD4^+^ T cell but not their subsets.

The LTNPs infected with HIV by blood products in China are unique, as the B′ subtype is predominant in these individuals [12] instead of the CRF_AE clade, which could lead to a faster CD4^+^ T cell decay in Chinese patients infected with HIV through sexual transmission [13]. In this study, we included 25 LTNPs infected with HIV by blood products and followed them up for approximately 4 years. Some of them experienced immunological progression, while the remaining individuals retained LTNP status during the follow-up period. We aimed to observe the dynamics of CD4^+^ T cell subsets and explore the determinants of CD4^+^ T cell decay in these specific LTNPs based on our longitudinal study. We believe this could improve clinical management for these individuals and extend our understanding of HIV pathogenesis.

## Methods

### Patients

This cohort study included 25 LTNPs who were all infected with HIV by blood products. Twenty-three of them were infected in the 1990s, and the remaining patients were infected in 2003, when cART was not widely available in China. Patients were followed up approximately every 6 months in the first three visits. Then, they were followed up annually for the last three visits. We observed the characteristics of immune cell profiles as well as the HIV RNA load in the clinics of the Department of Infectious Disease in Peking Union Medical College Hospital (PUMCH). Visit 1 was treated as the enrolment visit, and visit 6 or the last visit before cART initiation was considered as the latest visit.

### Definitions

LTNPs were defined by HIV seropositivity for at least 8 years, a stable CD4^+^ T cell count ≥400 cells/μL [14–16] and a lack of clinical symptoms in the absence of antiretroviral regimens. The loss of LTNP status was defined as a CD4^+^ T cell count of < 400 cells/μL at two consecutive measurements.

### Lymphocyte subsets and HIV-1 RNA measurement

Immunophenotyping of peripheral blood lymphocytes was conducted by three-colour flow cytometry (Epics XL flow cytometer; Beckman Coulter, USA) using commercially available monoclonal antibodies as previously described [17]. Freshly collected ethylenediaminetetraacetic acid and (EDTA)-anticoagulated whole blood was incubated and tested with a panel of monoclonal antibodies. The percentages and counts of the following lymphocyte subsets were measured, including CD3^−^CD19^+^ cells (B cells), CD3^−^CD16^+^CD56^+^ cells (NK cells), CD3^+^CD4^+^ cells (CD4^+^ T cells), CD3^+^CD8^+^ T cells (CD8^+^ T cells), and CD4^+^CD45RA^+^CD62L^+^ (naïve CD4^+^ T cells). The CD4^+^ T cell frequency was evaluated in total CD3 cells. The percentage of memory CD4^+^ T cells was counted by determining the difference between the percentage of CD4^+^ T cells and naïve CD4^+^ T cells.

Cell counts of lymphocyte subsets were determined using a dual-platform method with white blood cell counts and lymphocyte differentials obtained from routine blood tests of the same specimen.

Plasma HIV-1 RNA load was measured using the COBAS Ampliprep/TaqMan 48 real-time RT-PCR Test (Roche, CA, USA) according to the manufacturer’s instructions.

### Subtype analysis

The HIV Pol gene was amplified with a PrimeScript One Step RT-PCR Kit Ver.2 (TaKaRa, Dalian, China) and then sequenced. The PCR primers used for sequencing have been reported previously [13]. HIV-1 subtypes were then determined through the Recombinant Identification Program (http://www.hiv.lanl.gov/content/sequence/RIP/RIP.html) and confirmed by neighbour-joining phylogenetic analysis via sequence alignment of the Pol gene with reference sequences from the Los Alamos National Laboratory (http://www.hiv.lanl.gov/content/index).

### Measurements of anti-CMV IgG titer and plasma IL-32 alpha levels

Blood samples were collected from HIV-infected subjects to isolate plasma and then stored at − 80 °C until analysis. Plasma anti-CMV IgG levels were measured using enzyme immunoassay test kits (GenWay Biotech, San Diego, CA, USA), and IL-32 alpha levels were measured using the Quantikine ELISA Kit (Sino Biological, Cat # KIT11064) according to the manufacturer’s directions. The samples were read at 450 nm by Multiskan FC, and the concentration of target antigen was calculated.

### Ethics statement

The Institutional Review Board of PUMCH approved this study, and each participant provided written informed consent.

### Statistical analysis

Statistical analyses were performed using SPSS 23.0 (IBM Corp, Armonk, NY, United States). Descriptive statistics are presented as the median (M) values with interquartile ranges (IQRs). The Mann-Whitney U test was conducted for comparison of noncategorical variables. Categorical variables were compared by the Chi-squared test or Fisher’s exact test. Associations among continuous variables were tested using a nonparametric Spearman rank correlation test. A difference with a *p* value less than 0.05 was considered significant.

## Results

### Characteristics of the patients

In total, 25 LTNPs were identified in this study based on our definition. They had been infected with HIV for 12 (IQR: 11.5–14) years at enrolment. The route of infection was blood products, and 23 individuals involved the B′ subtype. Overall, 27.78, 5.56, 16.67, and 11.11% of patients had protective HLA-B*13, B*27, B*57 and B*58, respectively. The median CD4^+^ T cell count was 507 (IQR: 443–571) cells/μL, and the median HIV RNA load was 2.94 (IQR: 2.18–3.54) log_10_ cps/mL at study entry.

### Two clinical outcomes occurred during the follow-up period

Among the 25 patients, 6 individuals (decreased group, DG) experienced a loss of CD4^+^ T cell count during routine visits. One patient lost LTNP status in half a year of follow up. Two patients lost LTNP status in the first year and two were in the second year of follow up. The rest patient was in the fourth of follow up. The CD4^+^ T cell count was 237 (IQR: 213–320) cells/μL at the latest visit.

In total, 19 individuals retained LTNP status (non-decreased group, non-DG), their median CD4^+^ T cell count at the end of the follow-up was 561 (IQR: 470–626) cells/μL, and the median progression-free time was 16 (IQR: 16–18) years.

### Changes in naïve and memory CD4^+^ T cell counts in the two groups

At enrolment, the CD3^+^ T cell percentage was lower and the CD8^+^CD38^+^ subset was higher in the DG than the non-DG (*p* = 0.011, 0.03 respectively). However, there were no differences in the CD4^+^ T, CD8^+^ T, B, or NK cell counts (Table 1).

**Table 1:** Lymphocyte subsets of the two groups at enrolment

	DG	non-DG	
	(N = 6)	(N = 19)	***p***
CD19 + B counts (cells/uL)	164 (94–215)	160 (142–206)	0.799
CD19 + B percentage(%)	8.75 (5.68–13.6)	9.9 (7.8–12.9)	0.525
CD16CD56 + NK counts (cells/uL)	250 (153–315)	137 (82–178)	0.092
CD16CD56 + NK percentage (%)	13.5 (7.1–19.2)	8.8 (5.1–10.4)	0.143
CD3+ T counts (cells/uL)	1048 (924–1717)	1367 (1166–1536)	0.340
CD3+ T percentage (%)	71.2 (64.6–78.2)	79.9 (75.7–83.7)	0.011
CD3 + CD4 + T counts (cells/uL)	440 (432–616)	510 (472–572)	0.105
CD3 + CD8 + T counts (cells/uL)	574 (358–1184)	733 (570–856)	0.445
CD4 + CD45RA-(cells/uL)	225 (211–337)	291 (233–367)	0.181
CD4 + CD45RA + CD62L+(cells/uL)	212 (193–279)	188 (154–259)	0.408
CD8 + HLA-DR+/CD8 + (%)	36.9 (25.8–59.7)	32.6 (26.6–45.0)	0.408
CD8 + CD38/CD8 + (%)	62.1 (52.2–70.5)	48.0 (37.3–59.0)	0.03
CD4/CD8	0.76 (0.41–1.7)	0.75 (0.55–0.97)	0.949

**Fig. 1:**
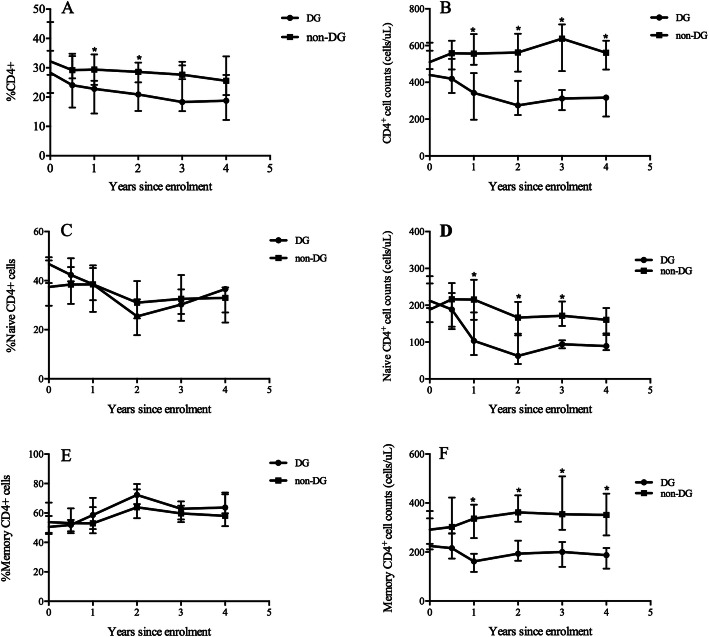
The dynamics of CD4^+^ T cell subsets during follow-up between DG and non-DG. The CD4^+^ T cell percentage decreased gradually in the DG(**a**). The CD4^+^ T cell count dropped significantly in the DG while it maintained the similar level with that at enrolment in the non-DG(**b**). The naïve CD4^+^ T cell percentage experienced a down trend(**c**) while memory CD4^+^ T cell percentage was comparable to that at enrolment (**e**). The naïve CD4^+^ T cell counts decreased significantly(**d**) while memory CD4^+^ T cell counts decayed slightly(**f**) in the DG

During the follow-up period, the CD4^+^ T cell count in the DG decreased gradually [the absolute cell count at the latest visit minus that at enrolment, delta CD4^+^ T cell count: -210 (IQR: − 383, − 117) cells/μL], but it was relatively stable in the non-DG [delta CD4^+^ T cell count: 48 (IQR: − 39, 129) cells/μL] (Fig. 1b). Both the naïve CD4^+^ T cell count and memory CD4^+^ T cell count fell significantly in the DG (Fig. 1d and f). In addition, the decrease in the naïve CD4^+^ T cell count [delta naïve = − 128 (IQR: − 196, − 107) cells/μL] was much greater than that in the memory CD4^+^ T cell count (delta memory = − 64 (IQR: − 182, − 25) cells/μL). However, the counts of these two CD4^+^ T cell subsets in the non-DG at enrolment were comparable to those at the latest visit [delta naive = − 21(IQR: − 86, 4) cells/μL, delta memory = 67 (IQR: 29, 122) cells/μL] (Fig. 1d and f, Fig. S1). No significant difference was observed in terms of CD4^+^ T cell percentage, naive CD4^+^ T cell percentage and memory CD4^+^ T cell percentage during follow-up period between the two groups (Fig. 1a, c, e).

**Fig. 2:**
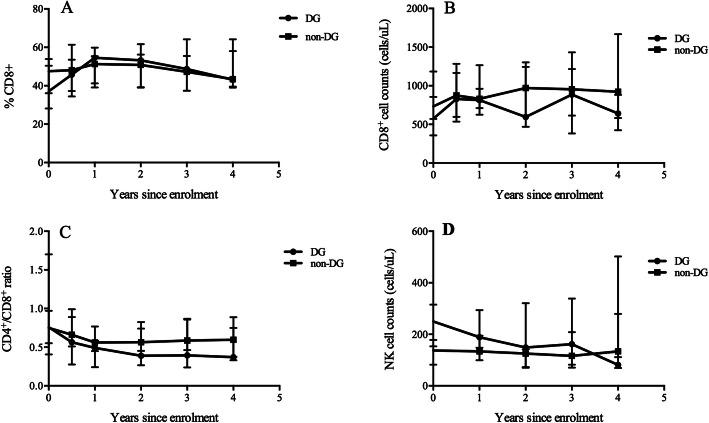
The fluctuations of lymphocyte subsets in the different visits. The CD8^+^ T cell percentage (**a**), CD8^+^ T cell counts (**b**), CD4^+^/CD8^+^ ratio (**c**) and NK cell counts (**d**) did not differ significantly between the two groups

The CD8^+^ T cell count in the non-DG was higher than that in the DG, but the difference was not significant (Fig. 2b) and the CD8^+^ percentage was comparable in two groups individuals (Fig. 2a). The same trend was observed for the CD4^+^/CD8^+^ ratio (Fig. 2c). The NK cell count was initially higher in the DG than in the non-DG; however, the difference gradually diminished, and the NK cell count in both groups was comparable at the end of follow-up (Fig. 2d). The B cell count progressively declined in the DG and further correlation analysis showed that the B cell count was positively associated with the CD4^+^ T cell count (r = 0.785, *p* < 0.001) (Fig. S2).

### Relationships between the expression of CD38 on CD8^+^ T cells and CD4^+^ T cell count

**Fig. 3:**
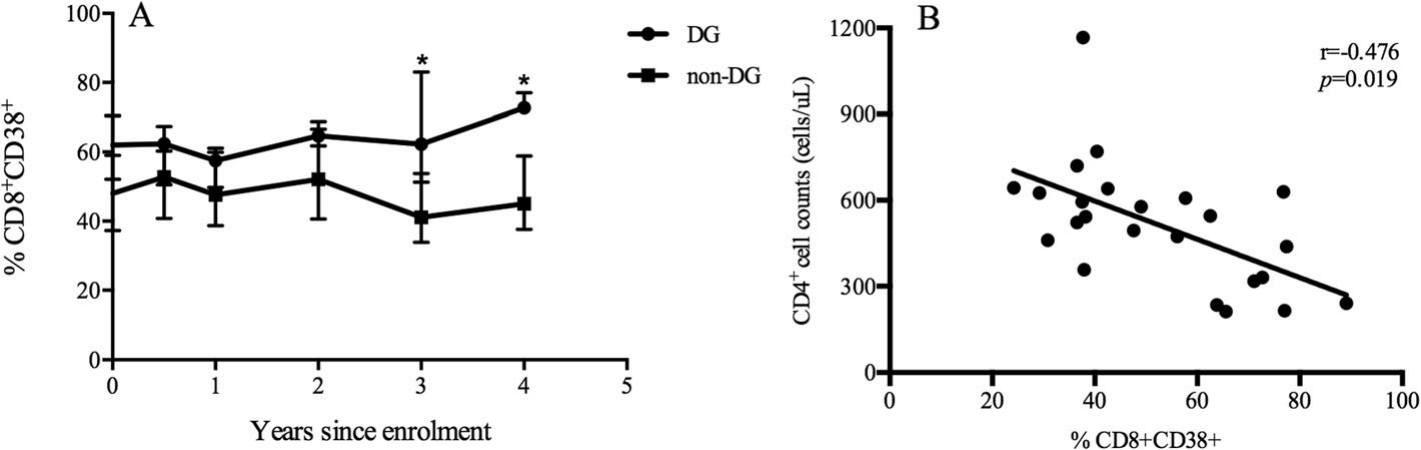
The activation subset of CD8^+^ T cell and its correlation with CD4^+^ T cell count. The dynamics of CD8^+^CD38^+^/CD8^+^ percentage during follow-up period was shown (**a**) and the percentage of the CD8^+^CD38^+^ subset was negatively associated with the CD4^+^ T cell count (**b**)

CD38 and HLA-DR are immune activation markers that are good predictors of disease progression in HIV-infected patients. Next, we analysed the dynamics of the CD8^+^CD38^+^ and CD8^+^HLA-DR^+^ percentages. The CD8^+^CD38^+^ percentage was higher in the DG at enrolment and rose gradually during follow-up (Fig. 3a). Additionally, the percentage of the CD8^+^CD38^+^ subset was negatively associated with the CD4^+^ T cell count (r = − 0.476, *p* = 0.019) (Fig. 3b). However, CD8^+^HLA-DR^+^ percentages did not differ significantly between the two groups (Data was not shown).

### The dynamics of the CD8^+^CD28^+^ percentage

The CD28 molecule has an important cellular function, providing a co-stimulatory signal for effector T lymphocyte activation, proliferation and cytokine production. We also analysed the fluctuation in the CD8^+^CD28^+^ percentage in both groups at four visits due to the data was not available for all time points. At the 4 years after enrolment, the CD8^+^CD28^+^ percentage in the DG exhibited a trend of being lower than that in the non-DG (Fig. S3A) and further correlation analysis showed that the CD8^+^CD28^+^ percentage was positively associated with the CD4^+^ T cell count (r = 0.448, *p* = 0.028) (Fig. S3B).

### Correlations between the HIV RNA level and the CD4^+^ T cell count

**Fig. 4:**
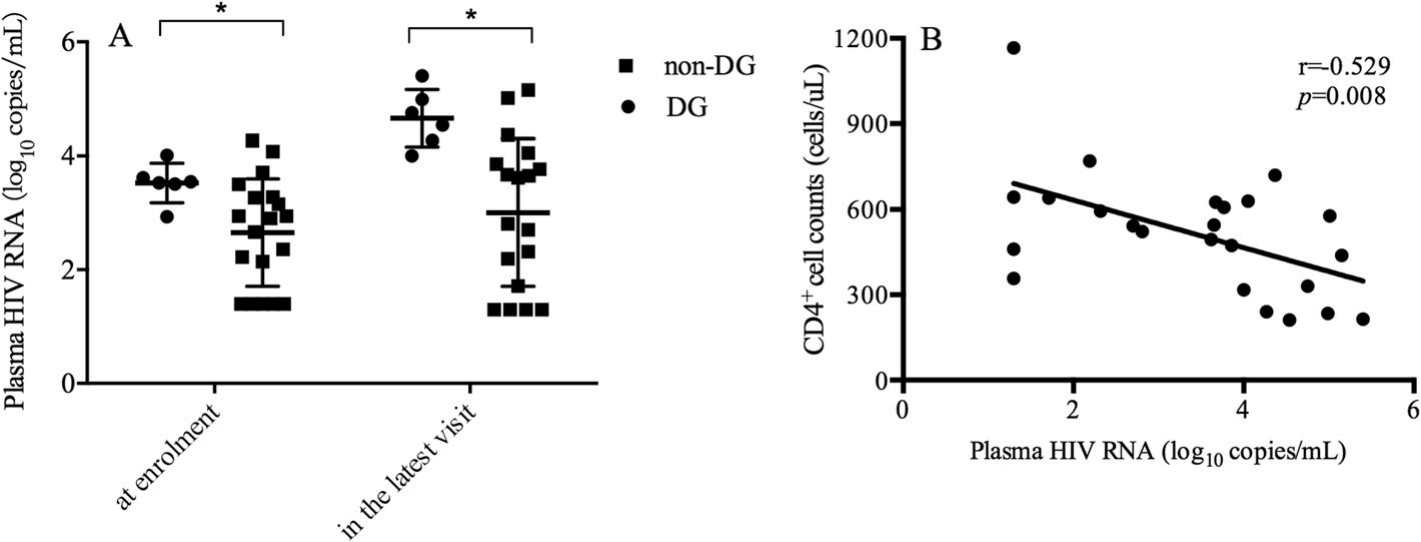
The plasma HIV RNA level and its correlation with CD4^+^ T cell count. The plasma HIV RNA level at enrollment and the latest visit between DG and non-DG was shown (**a**). Negative association was found between the plasma HIV RNA level and CD4 cell counts in LTNPs in the latest visit (**b**)

At enrolment, the viral load in the DG was higher than that in the non-DG (*p* = 0.03). Overall, 83.33% of individuals in the DG had > 2000 copies/mL HIV RNA while only 21.05% of individual in the non-DG had > 2000 copies/mL HIV RNA (Table 2). Moreover, the viral load was 4.64 (IQR: 4.20–5.09) log_10_ copies/mL in the DG, which was higher than that in the non-DG [3.22 (IQR: 1.61–3.91) log_10_ copies/mL] (*p* = 0.006) at the latest visit (Fig. 4a). The viral load increased by 1.15 (IQR: 0.67–1.54) log_10_ copies/mL (the viral load at the latest visit minus that at enrolment) in the DG, three folds that in the non-DG [0.33 (IQR: − 0.10, 0.84) log_10_ copies/mL] (*p* = 0.019). We further found a correlation between the viral load and the CD4^+^ T cell count in LTNPs (r = − 0.529, *p* = 0.008) (Fig. 4b).

**Table 2:** Characteristics of patients in the enrollment

	DG (***N*** = 6)	non-DG (***N*** = 19)	***p***
Median age at HIV diagnosis (years)	24 (20.5–31.5)	24 (22–27)	0.974
Sex			
Men	5 (83.33%)	12 (63.16%)	0.624
HIV infection time (years)	12 (8–15.25)	12 (12–14)	0.693
Protective HLA type^a^, n (%)	2 (50%)^b^	9 (64.29%)^c^	–
B*13	1 (25.0%)	4 (28.57%)	
B*27	0 (0%)	1 (7.14)	
B*57	0 (0%)	3 (21.43)	
B*58	1 (25.0%)	1 (7.14)	
HIV clade^d^			
B	4 (80.0%)	19 (100%)	0.208
CRF07_BC	1 (20.0%)	0 (0%)	
CD4 cell counts (cells/uL)	440 (432–616)	510 (472–572)	0.105
HIV RNA load (log_10_ copies/uL)	3.54 (3.37–3.72)	2.9 (1.40–3.28)	0.03
< 50 (copies/uL)	0 (0%)	5 (26.32%)	0.032
50–2000 (copies/uL)	1 (16.67%)	10 (52.63%)	
> 2000 (copies/uL)	5 (83.33%)	4 (21.05%)	

^a^: Protective gene were defined as HLA-B*57, HLA-B*27, HLA-B*13, HLA-B*58

^b^: 4 patients were tested for protective gene

^c^: 14 patients were tested for protective gene

^d^: 24 patients were tested for HIV subtype

### Changes of plasma anti-CMV IgG titer in the two groups

At enrolment, the median anti-CMV titer of 25 LTNPs was 7.6 (IQR: 5.37–11.35) IU/mL. There was no statistical difference between the DG and non-DG [6.67 (IQR: 5.0–9.79) vs 8.67 (IQR: 5.35–14.0), *p* = 0.426] (Fig. S4A). However, in the latest visit, we found that the median anti-CMV titer was significantly lower in the DG than in the non-DG [4.95 (IQR: 4.48–5.51) vs 10.13 (IQR: 7.33–11.79), *p* = 0.005] (Fig. S4A). Further analysis showed that CD4^+^ T cell count was not associated with the plasma levels of anti-CMV IgG (r = 0.306, *p* = 0.165). Moreover, we analyzed the association between CD4^+^ T cell counts and anti-CMV IgG titer in men and women. No significant difference was found (r = 0.167, *p* = 0.569), (r = 0.640, *p* = 0.087), respectively.

### The dynamics of plasma IL-32 alpha levels in the two groups

We examined how the plasma IL-32 alpha levels changed in the DG and non-DG. ELISA quantification of IL-32 alpha soluble protein in the plasma from non-DG subjects tended to be higher than that from the DG subjects at enrolment [2.35 (IQR: 2.12–2.57) log_10_ pg/mL vs 2.24 (IQR: 1.83–2.31) log_10_ pg/mL] and at the latest visit [2.1 (IQR: 1.91–2.35) log_10_ pg/mL vs 1.97 (IQR: 1.76–2.22) log_10_ pg/mL] (Fig. S4B). However, the difference did not achieve statistical significance (*p* = 0.121, *p* = 0.441, respectively).

## Discussion

Guidelines have recommended that HIV-infected patients should initiate cART as soon as possible, which means the identification of LTNPs will become increasingly challenging in the future. In our study, we enrolled 25 LTNPs who were infected with HIV by blood transmission, with the B′ subtype predominant, observing them for approximately 4 years. We found that 24.0% of LTNPs experienced CD4^+^ T cell count decay at 14.5 (IQR: 12.4–17.5) years following HIV infection. The decrease in the naïve CD4^+^ T cell count was more predominant than that in the memory CD4^+^ T cell count. Higher HIV RNA load levels were significantly associated with the decreased CD4^+^ T cell count and may be good predictor of CD4^+^ T cell count loss in LTNPs. Additionally, improved immune activation, lower CD8^+^CD28^+^ percentage and B cell count occurred in LTNPs with CD4^+^ T cell count decay. These results extend our understanding of the association between the clinical outcomes and HIV RNA loads levels as well as dynamics of immune cell profiles in LTNPs infected with HIV by blood products from China by long-term follow-up.

Literature including LTNPs who were infected with the B′ subtype by blood products revealed that HLA-B*13 was overrepresented among slow progressors in China [12]. Interestingly, our study also found that LTNPs had higher frequencies of HLA-B*13, but the distribution of the HLA-B*13, B*27 and B*57 was not significantly different between the DG and non-DG. The absence of protective effects of the HLA-B*13, B*27 and B*57 may be attributed to two reasons. First, the subjects in this project maintained LTNP status for at least 8 years; thus, it is reasonable to assume that they had the same genetic basis. This is similar to the lack of difference observed in the frequency of protective HLA alleles among ECs, virological controllers and non-virological controllers [9], all of whom showed slow progression. Second, it was reported that epitopes mutations occurred in HLA (human leukocyte antigen)-directed immune responses in HIV-infected patients, which could further diminish the protective effect [18, 19].

A previous study showed that the percentages of naïve CD4^+^ T cells was not significantly different between LTNPs and TPs, which was comparable to that in HIV-negative persons [20]. However, the naïve CD4^+^ T cell count was significantly lower in ECs with reduced CD4^+^ T cell counts compared to that in ECs with normal CD4^+^ T cell counts [21]. In this study, we additionally found that the decrease of naïve CD4^+^ T cell counts was more dominant than that of memory CD4^+^ T cell counts during the follow-up period in LTNPs with CD4 ^+^ T cell count decay, indicating an important role of naïve CD4^+^ T cell count in the maintenance of CD4^+^ T cell homeostasis.

HIV replication particularly occured in memory CD4^+^ T cells, leading to the loss of memory CD4^+^ T cells in typical progressors (TPs) [22]. In our study, HIV RNA level was significantly higher in DG at enrolment and the latest visit. On one hand, the increasing HIV RNA replication may result in more memory CD4^+^ T cell loss. On the other hand, study showed that in HIV-infected patients, HIV RNA level was weakly associated with central memory CD4^+^ T cell and was strongly associated with the proliferation of effector memory cells in CD4^+^ T cell [23]. Thus, the proliferation of memory CD4^+^ T cell may compensate for its loss in some extent, which contributed to the less dominant decrease in memory CD4^+^ T cell count in DG.

Persistent HIV RNA replication also results in systematic immune activation, which could contribute to progressive CD4^+^ T cell count depletion in TPs [24]. HIV controllers are considered to have low levels of T cell activation [25], meaning immune activation plays a key role in the disease progression of HIV-infected individuals. CD38 is an activation marker, and the elevated expression of CD38 has a strong relationship with activation and cell aging. In our study, the CD8^+^CD38^+^ percentage in the DG was higher than that in the non-DG at enrolment and increased steadily throughout the follow-up period. Moreover, a significantly inverse correlation between the CD8^+^CD38^+^ percentage and CD4^+^ T cell count was observed, which was similar to the findings of a previous study [26]. Moreover, increased HIV RNA levels and immune activation in the DG may have a mutual effect that is also associated with systemic inflammation. These factors may facilitate apoptosis in CD4^+^ T cells, leading to a decrease in the CD4^+^ T cell count. Additionally, immune activation leads to low thymic output in HIV-infected patients [27], possibly explaining why patients with DG experience large decreases in the naïve CD4^+^ T cell count. In summary, these results suggest that continuous immune activation might contribute to disease progression in some LTNPs, as it does in TPs.

The CD28 molecule provides secondary signals that are important for the activation of naïve T cells [28], playing a key role in specific T cell responses. Repeated antigenic exposure contributes to a reduction in the proportion of CD8^+^CD28^+^ cells and an increase in the proportion of CD8^+^CD28^−^ cells [29]. HIV-specific T cell responses are effective in suppressing viral replication [30]. Improving viral load in our study may lead to elevated HIV-specific T cell responses to suppress HIV RNA replication. During this progression, more CD8^+^CD28^+^ subsets may convert to CD8^+^CD28^−^ cells, leading to the decrease of the CD8^+^CD28^+^ subset.

B cells play an important role in producing effective antibodies against HIV-infected cells; however, memory B cell defects have been widely reported in HIV infection, which is likely to reflect defects in CD4^+^ T cell help [31]. In our study, we observed that B cell count decreased gradually with decreasing CD4^+^ T cell count in the DG. This pattern may indicate the expansion of immature B cells, which are highly susceptible to intrinsic apoptosis and may contribute to the depletion of B cells [32, 33]. Interestingly, a study conducted in patients with primary HIV infection showed a similar finding: the B cell count correlated positively with the CD4^+^ T cell count during a 12-month follow-up, indicating that the B cell count might be linked with disease progression [34].

A previous study showed that anti-CMV IgG levels were associated with the occurrence of cardiovascular disease in HIV-infected women [35]. This may be the result of chronic inflammation in HIV-infected individuals. Notably, annual CD4^+^ T cell count changes were lower in those with higher anti-CMV IgG levels in HIV ECs [8]. However, another study reported that anti-CMV IgG levels were not associated with CD4^+^ T cell counts in HIV-infected men [36]. In our study, we found that anti-CMV IgG titer was lower in the DG than in the non-DG in the latest visit, but no correlation was found between anti-CMV IgG and CD4^+^ T cell counts, similar to a previous study [36]. Based on the dynamics of B cell count in our study, the change of anti-CMV IgG titer may be correlated with the decreased B cell count.

IL-32 alpha was shown to be the less inflammatory isoform [37], and previous research found that IL-32 alpha tended to be higher in ECs who had HIV RNA < 50 copies/mL and CD4^+^ T counts > 500 cells/mm^3^ compared to TPs [9]. Our results showed that there was a tendency for higher IL-32 alpha from non-DG subjects than DG subjects. These results indicated that immune inflammation may play an important role in maintaining stable CD4^+^ T cell counts. Future research could explore the impact of other isoforms of IL-32 on CD4^+^ T cell count trajectory.

Some limitations exist in this study. First, the sample size was small, but we followed up these patients for approximately four years and observed the dynamics of CD4^+^ T cell subsets, which made our results more convinced. Second, uniform definitions of LTNP have not been formulated. The threshold of CD4^+^ T cell counts for LTNP was 500 cells/μL, while others have employed a cutoff of 400 cells/μL. We defined LTNP as having a CD4^+^ T cell count > 400 cells/μL, which has been used less frequently. Finally, we did not have access to the counts of lymphocyte subsets or levels of HIV RNA at the time that the LTNPs were diagnosed with HIV infection. Thus, we could not determine whether these values affected disease progression. In spite of these limitations, our study shows that immunological progression mainly consisted of the naïve CD4^+^ T cell count loss in LTNPs infected with HIV by blood products. Moreover, increasing HIV RNA load may contribute to the immunological progression in these patients.

## Conclusion

In conclusion, our longitudinal study proposes that high HIV RNA load is probably associated with immunological progression, which is characterized by the significant decrease of naïve CD4^+^ T cell counts in LTNPs harbouring the B′ subtype. These results may be beneficial for managing LTNPs and also provide important insight into HIV pathogenesis.

## Supplementary Information

**Additional file 1: Fig. S1.** The change in subsets of CD4 cell counts between the two groups

**Additional file 2: Fig. S2.** The B cell count and its correlation with CD4^+^ T cell count. The B cell count declined at first, then maintained relatively stable in the DG while in the non-DG, B cell count was stable before experiencing some fluctuations (A). The B cell count was positively associated with the CD4^+^ T cell count (B).

**Additional file 3: Fig. S3.** The CD8^+^CD28^+^/CD8^+^ percentage and its correlations with CD4^+^ T cell counts. The CD8^+^CD28^+^/CD8^+^ percentage in two groups dropped at first, then it was stable in the DG while it increased to the similar level in the first years since enrolment in the non-DG (A). The CD8^+^CD28^+^/CD8^+^ percentage was positively associated with the CD4^+^ T cell count (B)

**Additional file 4: Fig. S4.** The changes of plasma anti-CMV titer and IL-32 alpha levels between two groups at enrolment and in the latest visit. No statistical difference between the two groups at enrolment while in the latest visit, the median anti-CMV titer was significantly lower in the DG than in the non-DG (A). No statistical significance was found between the two groups (B).

**Additional file 5.**

## Data Availability

Datasets used in this analysis are available from the corresponding author upon request.

## Abbreviations

cART: Combined antiretroviral therapy
HIV: Human immunodeficiency virus
LTNPs: Long-term non-progressors
DG: Decreased CD4^+^ T cell count group
SG: Stable CD4+ T cell count group
PUMCH: Peking Union Medical College Hospital
