# Supplementary figures and images for: Naïve CD4+ cell counts significantly decay and high HIV RNA levels contribute to immunological progression in long-term non-progressors infected with HIV by blood products: a cohort study
Source: BMC Immunol. 2021 Jun 3;22:36. doi: 10.1186/s12865-021-00426-8 (PMC8173962; doi:10.1186/s12865-021-00426-8)

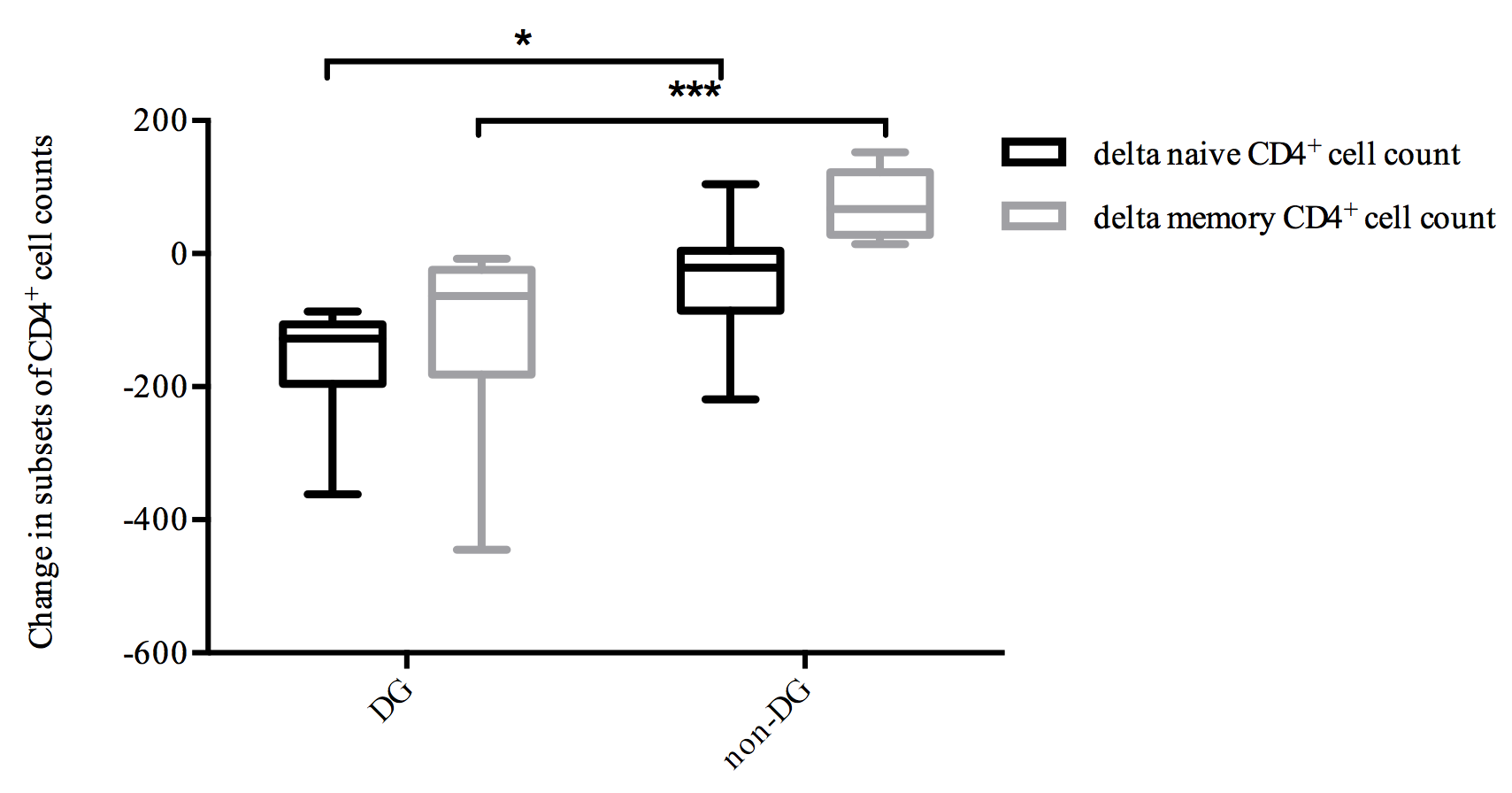

Supplement: Supplementary file 1 — Additional file 1: Fig. S1. The change in subsets of CD4 cell counts between the two groups [file 12865_2021_426_MOESM1_ESM.tiff]

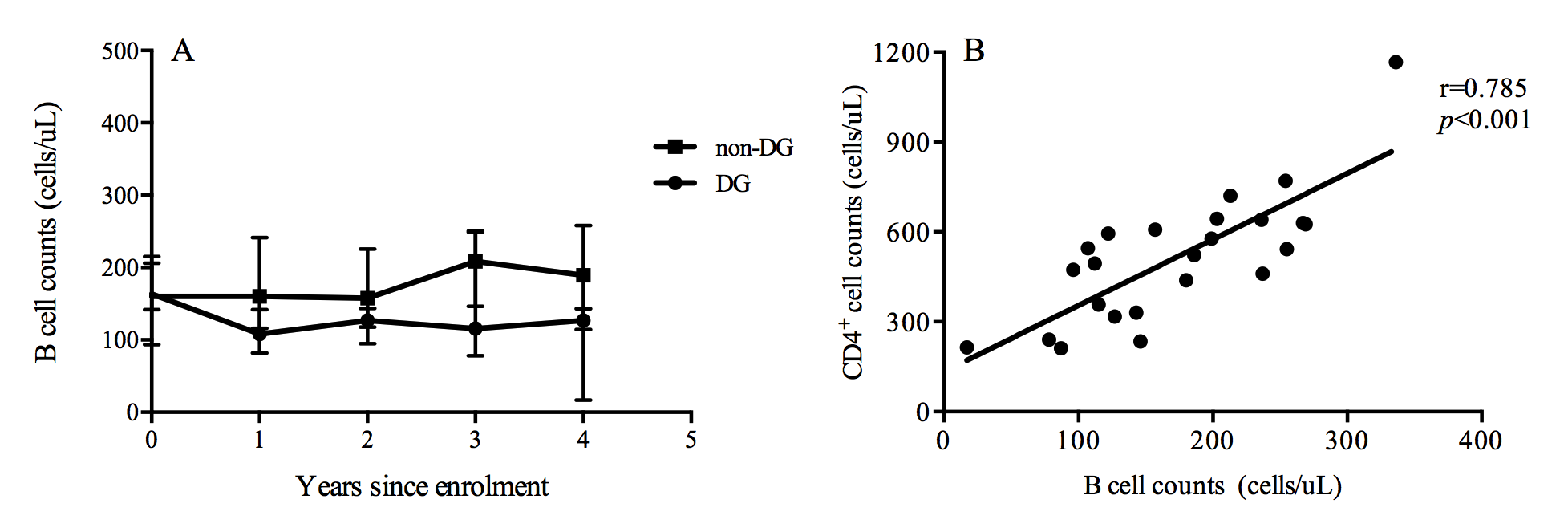

Supplement: Supplementary file 2 — Additional file 2: Fig. S2. The B cell count and its correlation with CD4+ T cell count. The B cell count declined at first, then maintained relatively stable in the DG while in the non-DG, B cell count was stable before experiencing some fluctuations (A). The B cell count was positively associated with the CD4+ T cell count (B). [file 12865_2021_426_MOESM2_ESM.tiff]

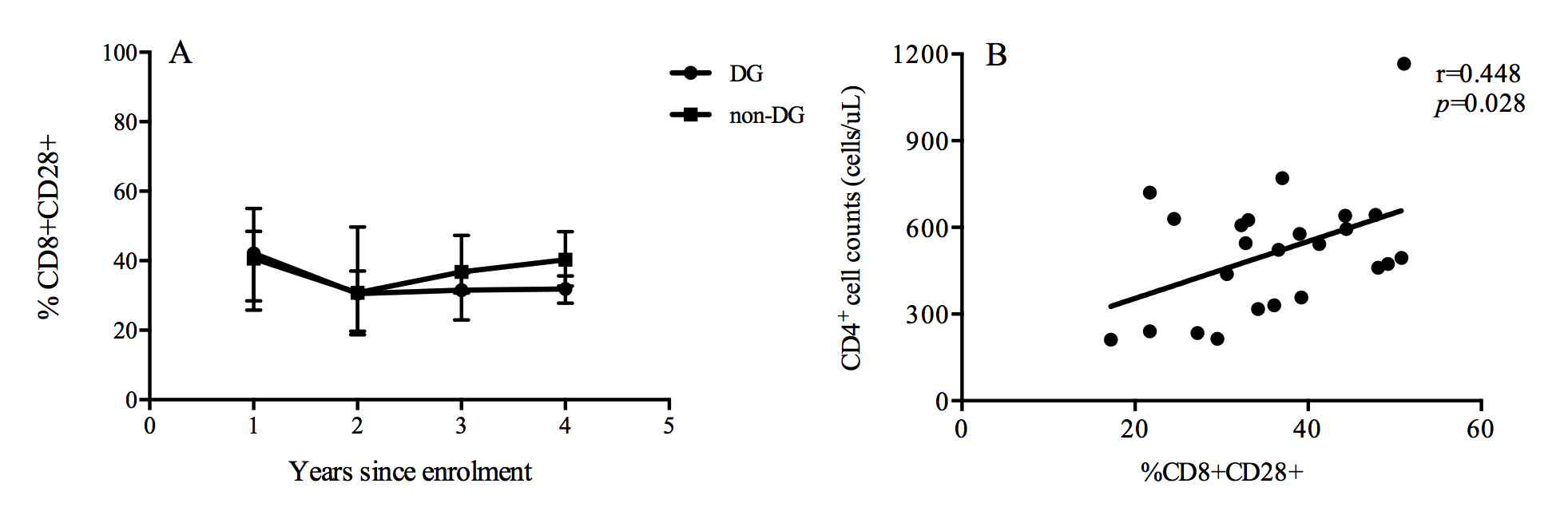

Supplement: Supplementary file 3 — Additional file 3: Fig. S3. The CD8+CD28+/CD8+ percentage and its correlations with CD4+ T cell counts. The CD8+CD28+/CD8+ percentage in two groups dropped at first, then it was stable in the DG while it increased to the similar level in the first years since enrolment in the non-DG (A). The CD8+CD28+/CD8+ percentage was positively associated with the CD4+ T cell count (B) [file 12865_2021_426_MOESM3_ESM.tiff]

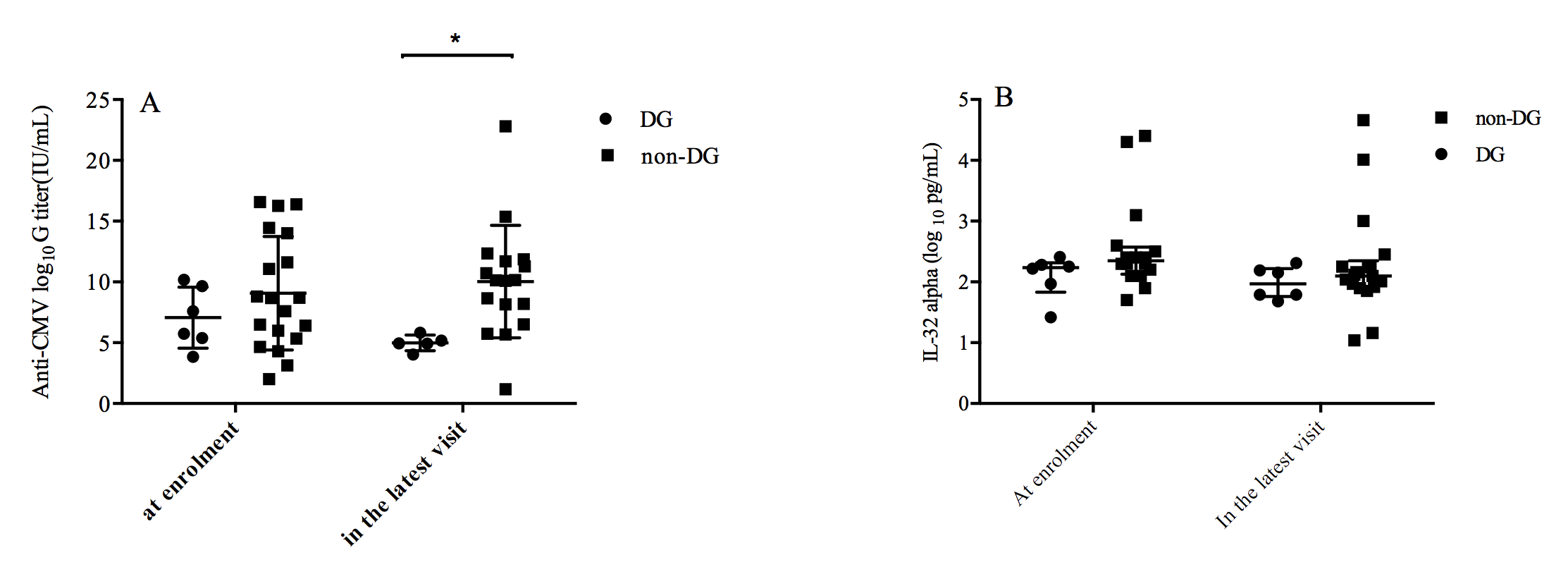

Supplement: Supplementary file 4 — Additional file 4: Fig. S4. The changes of plasma anti-CMV titer and IL-32 alpha levels between two groups at enrolment and in the latest visit. No statistical difference between the two groups at enrolment while in the latest visit, the median anti-CMV titer was significantly lower in the DG than in the non-DG (A). No statistical significance was found between the two groups (B). [file 12865_2021_426_MOESM4_ESM.tiff]

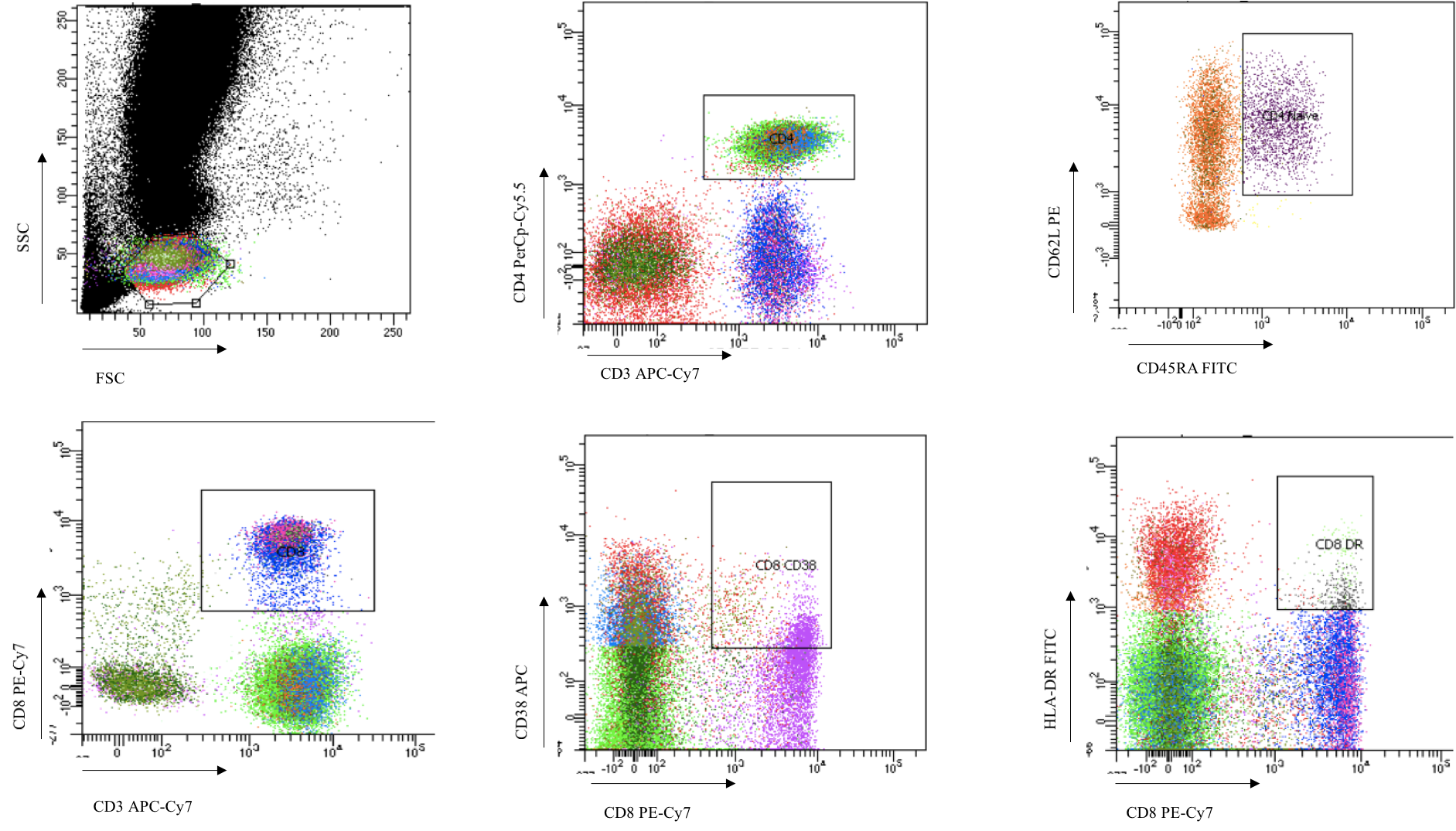

Supplement: Supplementary file 5 — Additional file 5. [file 12865_2021_426_MOESM5_ESM.tiff]
